# From Doubt to Confidence—Overcoming Fraudulent Submissions by Bots and Other Takers of a Web-Based Survey

**DOI:** 10.2196/60184

**Published:** 2024-12-16

**Authors:** Jeffrey J Hardesty, Elizabeth Crespi, Joshua K Sinamo, Qinghua Nian, Alison Breland, Thomas Eissenberg, Ryan David Kennedy, Joanna E Cohen

**Affiliations:** 1 Institute for Global Tobacco Control Department of Health, Behavior and Society Johns Hopkins University Baltimore, MD United States; 2 Center for the Study of Tobacco Products Department of Psychology Virginia Commonwealth University Richmond, VA United States

**Keywords:** fake data, recruitment, online survey, internet survey, challenges, data integrity, data quality, e-cigs, tobacco control, longitudinal survey, web-based survey, e-cigarette, vaping, smoking, smoke, cessation, prevalence, data collection, United States, US, adult, VAPER, Vaping and Patterns of E-cigarette Use Research

## Abstract

In 2019, we launched a web-based longitudinal survey of adults who frequently use e-cigarettes, called the Vaping and Patterns of E-cigarette Use Research (VAPER) Study. The initial attempt to collect survey data failed due to fraudulent survey submissions, likely submitted by survey bots and other survey takers. This paper chronicles the journey from that setback to the successful completion of 5 waves of data collection. The section “Naïve Beginnings” examines the study preparation phase, identifying the events, decisions, and assumptions that contributed to the failure (eg, allowing anonymous survey takers to submit surveys and overreliance on a third-party’s proprietary fraud detection tool to identify participants attempting to submit multiple surveys). “A 5-Alarm Fire and Subsequent Investigation” summarizes the warning signs that suggested fraudulent survey submissions had compromised the data integrity after the initial survey launched (eg, an unanticipated acceleration in recruitment and a voicemail alleging fraudulent receipt of multiple gift codes). This section also covers the investigation process, along with conclusions regarding how the methodology was exploited (eg, clearing cookies and using virtual private networks) and the extent of the issue (ie, only 363/1624, 22.4% of the survey completions were likely valid). “Building More Resilient Methodology” details the vulnerabilities and threats that likely compromised the initial survey attempt (eg, anonymity and survey bots); the corresponding mitigation strategies and their benefits and limitations (eg, personal record verification platforms, IP address matching, virtual private network detection services, and CAPTCHA [Completely Automated Public Turing test to tell Computers and Humans Apart]); and the array of strategies that were implemented in future survey attempts. “Staying Vigilant” recounts the identification and management of an additional threat that emerged despite the implementation of an array of mitigation strategies, underscoring the need for ongoing vigilance and adaptability. While the precise nature of the threat remains unknown, the evidence suggested multiple fraudulent surveys were submitted by a single or connected entities, who likely did not possess e-cigarettes. To mitigate the chance of reoccurrence, participants were required to submit an authentic photo of their most used e-cigarette. Finally, in “Reflection 4 Years Later,” we share insights after completing 5 waves of data collection without additional threats or vulnerabilities uncovered that necessitated the application of further mitigation strategies. Reflections include reasons for confidence in the data’s integrity, the scalability and cost-effectiveness of the study protocols, and the potential introduction of sampling bias through recruitment and mitigation strategies. By sharing our journey, we aim to provide valuable insights for researchers facing similar challenges with web-based surveys and those seeking to minimize such challenges a priori. Our experiences highlight the importance of proactive measures, continuous monitoring, and adaptive problem-solving to ensure the integrity of data collected from participants recruited from web-based platforms.

## Introduction

Researchers increasingly use web-based surveys for data collection, offering them multiple options to source participants [1]. Companies, such as Qualtrics, offer web-based panels of possible survey participants, who are preidentified persons willing to respond to web questionnaires. Other companies, such as Amazon Mechanical Turk, offer crowdsourcing platforms that pair “workers” with paid web-based tasks that require effort from multiple people, including web-based surveys. In addition, researchers can create an original sample of web-based survey participants through recruitment strategies such as ads on social media platforms.

Each of the 3 options for sourcing participants has relative strengths and trade-offs in terms of the required effort from the research team, cost, speed of recruitment, data quality, maximum sample size, and representativeness of the target sample. For example, those building a web-based sample using a company-provided web panel or a crowdsource platform may find these ready-to-use options for sourcing participants result in studies achieving their desired sample size more quickly and at a lower cost as compared with an original sample recruited via social media ads or similar web-based recruitment approach. However, web-based panels and crowdsourcing platforms are not all created equally. Some companies provide researchers access to their web panel and oversee sampling and survey administration for a cost, while crowdsourcing platforms are more self-service and only provide access to their “workers.” Furthermore, Peer et al [2] found that data quality may vary among web panels and crowdsourcing platforms, and others have reported poor data integrity due to participant misrepresentation of credentials after using a well-known company’s subcontracted web panels [3]. Researchers expecting to recruit lower-prevalence populations may also find that the attainable sample size, pace of recruitment, or representativeness from existing web-based panels and crowdsourcing platforms are inadequate for their study aims [4].

In contrast to those using a company-provided web panel or crowdsource platform, researchers who are interested in building a web-based original sample must plan, build, test, and implement recruitment and data collection methods and related strategies, which may result in slower recruitment and higher costs. However, these bespoke solutions may allow researchers to recruit lower-prevalence populations more efficiently once established, to achieve a more representative sample, or to improve data quality. Achieving these benefits requires not only expertise in survey sampling techniques but also proficiency in mitigating risk to data integrity [5-7]. Importantly, the latter includes minimizing fraudulent survey submissions from individuals who aim to deceive researchers, typically to receive incentives [5,8-10]. Pozzar et al [11] conducted a data integrity experiment and found that as much as 94.5% of their sample, which was recruited by social media and intended to be English-speaking US adults diagnosed with ovarian cancer within the last 12 months, was fraudulent. This occurred despite implementing practices consistent with lessons learned from previously published research and seeking advice from their institution’s Research Electronic Data Capture (REDCap) administrators and survey research core (eg, eligibility criteria were not apparent on promotions, CAPTCHA [Completely Automated Public Turing test to tell Computers and Humans Apart], and a hidden questionnaire item visible only to survey bots) [11]. Others such as Pratt-Chapman et al [6] aimed to recruit cancer survivors through social media and removed three-fourths of their initial sample, despite using CAPTCHA, a hidden questionnaire item, instructions to type a specific word, and other strategies to mitigate the impact of survey bots that automate the completion of multiple surveys. Griffin et al [12] had a similar experience with social media and professional listserve recruitment of people who identify as LGBTQ+ (lesbian, gay, bisexual, transgender, queer) and removed 61.8% of their initial survey responses, despite using Qualtrics’ “Prevent Ballot Box Stuffing” feature (prevents multiple submissions from one individual by placing a cookie in the browser) and survey bot detection features [13]. These recent examples suggest that recommendations from the literature and other sources for addressing fraudulent survey submissions may be insufficient, improperly applied, or both, necessitating that researchers continue to share their failures and successes so that others may learn from their experiences.

In 2019, we launched the Vaping and Patterns of E-cigarette Use Research (VAPER) Study, which has been described in detail elsewhere [14]. Participants were surveyed to understand better how their product use may change in response to regulations. We recruited a web-based original sample of a lower-prevalence population, adults who frequently use e-cigarettes (5+ days per week), to join a longitudinal web-based survey. Over the next 4 years, we experienced a failed survey attempt, learned about the threats and vulnerabilities that likely led to the fraudulent survey submissions that compromised the data integrity, learned about the benefits and limitations of risk mitigation strategies, implemented an effective array of mitigation strategies, and, ultimately, achieved the desired sample size and ended with reasonable confidence in the data integrity. This paper has 5 aims. First, we aim to summarize the events, decisions, and assumptions during the study preparation phase that likely led to the failed initial survey attempt (“Naïve Beginnings”). Second, we present the warning signs that suggested fraudulent survey submissions may have compromised the data integrity after the initial survey was launched, and the results from the subsequent investigation into the extent of the issue and how the methodology was exploited (“A 5-Alarm Fire and Subsequent Investigation”). Third, we describe the threats and vulnerabilities that likely led to the fraudulent survey submissions during the initial survey attempt (with a focus on anonymity and survey bots), the corresponding mitigation strategies and their benefits and limitations, and the array of strategies that were implemented in future survey attempts (“Building More Resilient Methodology”). Fourth, we detail our experience identifying and addressing another threat and vulnerability that led to suspicious survey submissions despite various mitigation strategies in place after restarting wave 1 (“Staying Vigilant”). Last, we share reflections after completing 5 waves of data collection with no additional threats or vulnerabilities uncovered that warranted the application of additional mitigation strategies (“Reflections 4 Years Later”).

### Ethical Considerations

Ethical approval was obtained from the IRB of the Virginia Commonwealth University (approval HM20015004; date: May 14, 2019), with the Johns Hopkins Bloomberg School of Public Health IRB relying on the IRB of Virginia Commonwealth University as the IRB of record (IRB9277).

Participants were directed to read the written consent form on a web page and click “Agree” or “Do not agree” in response to a prompt asking, “Do you consent to answering the screening and survey questions?” Participants were also provided with the opportunity to re-read the consent form prior to each follow-up questionnaire. A waiver of documentation of signatures was received from the IRB. As part of the informed consent, participants consented to allow the use of their nonidentifying data in future research studies and distribution of their nonidentifying data to the study sponsor or another researcher for future studies without additional informed consent.

During the study, risk for loss of confidentiality was mitigated by securely storing documentation containing participant-identifying information on university servers with restricted access. A Certificate of Confidentiality was also obtained from the National Institutes of Health to help keep participant data private (eg, to prevent a court from obtaining participant data in a court case). Upon study completion, all documentation containing identifying information was deidentified.

Participants who completed the survey during the failed initial survey attempt received a $10 gift code from a company of their choice as compensation. After implementing risk mitigation strategies, participants in waves 1-5 received Amazon gift codes worth US $10-$30, with the amount varying based on the survey wave, whether it was a baseline or follow-up survey, and the subset of questions completed.

## Naïve Beginnings

The VAPER Study recruited adults aged 21 years and older who used e-cigarettes at least 5 days per week. This was a lower prevalence population, with 2.3% of US adults using e-cigarettes daily and 5.1% of adults using in the past 30 days in 2020 [15]. A sample of at least 1200 participants was required to adequately power the VAPER Study’s initial hypotheses, after adjusting for an anticipated loss to follow-up rate of 25% and assuming an effect size of 10% (for *t* tests to detect differences between 2 dependent means using a 2-tailed test), Cronbach α<0.05, and power of 0.85 [14]. Additionally, a subset of the sample was intended to attend an in-person laboratory visit for puff topography measurements (measurements of the duration, volume, and frequency of puffs taken during the use of an e-cigarette), which required limiting recruitment to Richmond, Virginia; Columbus, Ohio; and Los Angeles, California, where collaborating laboratory partners were located.

We found that no existing web-based panel or crowdsourcing platform available during 2019 could support the study due to sample size considerations and the limited catchment areas, necessitating that we build a web-based original sample. We collaborated with a university-based survey research center in the United States with experience in web-based recruitment and survey data collection to build the survey using a questionnaire provided by our team, implement a Facebook- and Instagram-based recruitment strategy (specific platforms were recommended by the research center), and distribute incentives.

The survey was custom-built on a proprietary platform. Data storage protections primarily included network security measures (eg, partitioned network storage to mitigate the potential for data loss and to administer appropriate permissions) and integrity verification (eg, Varonis DatAdvantage to report on authorized and unauthorized changes to file server data). The research center also used a third-party’s proprietary fraud detection tool to identify participants attempting to take the survey multiple times. At the time of its inclusion, the tool functioned by monitoring and modeling metadata and behaviors to generate a fraud profile score. In addition, another third-party tool was used to streamline the delivery of incentives to participants in the form of US $10 gift codes from a company of their choosing. Each survey was intended to last about 20 minutes.

To help increase the pace of recruitment, anonymous survey taking was permitted, allowing participants to register for the survey by signing in with a Gmail or Facebook account or by creating an account with any email address. These joint decisions were informed by the research center’s previous experiences with web-based recruitment and web-based survey data collection and the assumption that the third-party detection tool intended to prevent multiple survey attempts would minimize fraudulent survey submissions and preserve data integrity.

## A 5-Alarm Fire and Subsequent Investigation

The target population’s lower prevalence combined with a limited catchment area resulted in modest expectations for the pace of recruitment. During the first 4 weeks of recruitment, each city yielded 1-10 participants per day, which was consistent with these expectations. However, approximately 1 month after recruitment began, the pace of recruitment accelerated from 1-10 to 11-124 submissions per day per city. Upon receiving a participant’s voicemail message alleging fraudulent receipt of multiple gift codes, concerns arose about the data integrity. Consequently, recruitment, data collection, and incentive delivery were halted, and a 2-month investigation of 1624 survey submissions commenced to determine how participants had exploited the methodology and the approximate number of fraudulent survey submissions.

The investigation revealed that participants likely had circumvented the mitigation strategies by clearing cookies, using new browsers or devices, using virtual private networks (VPNs), discerning a pattern in survey URL generation, and interfering with the third-party fraud detection tool. As identifiable data were not collected, determining the precise extent of the issues was not feasible. As a result, a liberal approach was adopted to identify fraudulent survey submissions using a set of criteria. These criteria included, but were not limited to, short completion times, surveys taken without first clicking a social media ad, duplicate account information (eg, same or similar email addresses), and the other indicators, as detailed in Textbox 1.

Based on these criteria, it was determined that only 363 of the 1624 survey completions were likely valid, representing 22.4% of the total. Due to the imprecision of labeling participants as “likely valid” and the possibility of inviting participants who submitted fraudulent surveys to another survey, these data were not used for analysis and the participants were not invited to participate in future survey attempts.

**Key criteria for identifying fraudulent survey submissions used during the investigation of the failed initial survey attempt.**

**Criteria**

- Short completion times
- Surveys taken without first clicking a social media ad
- Duplicate account information (eg, same or similar email addresses)
- Unusually high amount of “don’t know” and “prefer not to answer” responses (2 SDs above the mean)
- Third-party fraud detection tool scores suggesting multiple surveys were submitted by a participant
- Duplicate photos submitted across survey submissions (optional photos of their e-cigarette device were requested during the survey)
- Use of non-English alphabet (eg, Cyrillic alphabet)
- Ad-location mismatches (eg, an ad was for Los Angeles, California, and the survey was taken in Boston, Massachusetts)
- Unusually high agreement between responses across survey submissions suggesting multiple surveys were submitted by a participant (2 SDs above the mean)
- Inconsistent responses to questionnaire items (eg, inconsistent device type and brand)

## Building More Resilient Methodology

### Overview

Prior to resuming data collection, we reviewed the literature and sought guidance from independent data authentication experts. The experts consistently indicated that while fraudulent survey submission and data integrity issues cannot be completely prevented, they can be mitigated through a combination of strategies. Their recommendations included addressing anonymity, using CAPTCHA to mitigate the impact of survey bots, shortening data collection windows, and randomizing survey URLs generated. In this section, we describe the threats and vulnerabilities that likely led to the fraudulent survey submissions during the initial survey attempt (with a focus on anonymity and survey bots), the corresponding mitigation strategies and their benefits and limitations, and the array of strategies that were implemented in future survey attempts.

### Anonymity

Anonymity may be appropriate in certain study designs [16], but surveys that recruit anonymous participants and collect data on the internet are vulnerable [7,17] and can be compromised by a single or group of individuals with the intent to deceive. Anonymity allows for the unfettered creation and use of survey bots and multiple email addresses, phone numbers, IP addresses (eg, via VPNs), browsers, and devices to complete one or multiple fraudulent survey submissions. This situation arises because anonymous fraudulent survey submissions are more difficult to detect with certainty and anonymous participants can submit additional fraudulent surveys using the same or different strategies even after detection and removal from the dataset.

### Anonymity: Mitigation Strategies and Their Benefits and Limitations

Using a personal record verification platform can mitigate anonymity concerns significantly. To use these services, researchers must collect each participant’s name, date of birth, and residential mailing address and cross-reference these data against information about the participant on the platform. These 3 forms of verified personally identifiable data are a powerful tool for determining whether a survey likely was submitted by a unique and real participant, considering that their unverified equivalents can be fabricated and functioning email addresses and phone numbers can be generated and authenticated by individuals who aim to deceive researchers. Nevertheless, this strategy is not foolproof. Limitations include the following: (1) post office (PO) box addresses may not be tracked by the personal record verification platform; (2) personally identifiable data can be obtained illegally (eg, on the dark web, an encrypted subsection on the internet that is not indexed by a typical search engine and frequently used to conduct illegal activities) [18]; (3) participants can own or live at multiple addresses; (4) participants can use the address of a family member, friend, or neighbor; (5) personal record verification platforms have imperfect datasets (eg, participants move and the dataset may not have updated); and (6) participants can make honest mistakes (eg, typos or provide nicknames), resulting in mismatched data at no fault of the participant.

In addition, IP address matching can be used to identify instances where an individual has submitted multiple surveys, and IP address lookup tools can be used to verify participants’ approximate geographic location. However, the utility of these 2 strategies is limited because IP addresses were not invented to identify internet users [19]. Rather, they were created to identify devices on a network that transmit data [19]. This key distinction is important as there are tools available that can be used to manipulate IP addresses. Such tools include VPNs, which are web-based applications that allow users to access the internet through remote servers that may be located anywhere in the world, providing them with access to many IP addresses that can give researchers the false impression they are physically located in a specific country or subnational location [20]. VPNs pose a particular challenge to data integrity given their increasingly common use; a nationally representative survey conducted by a VPN provider suggests that 33.0% of Americans in 2023 use a VPN compared with 24.3% in 2022 [21]. Beyond VPNs, networks of proxy servers, such as Tor via Tor Browser, can be used to route internet traffic through more than one intermediary server [22], effectively hiding its users’ real IP addresses. Other approaches to changing a device’s IP address include resetting a router [23], using the same device on a different Wi-Fi network [24], and switching a mobile phone from a Wi-Fi network to a cellular network [25].

VPN detection services are an available tool that can assess whether IP addresses are likely associated with VPNs [26]. These services use various detection methods, with the simplest and most effective method being IP address blocklists, which match user IP addresses against known VPN-associated IP addresses. Other less effective methods include deep packet inspection, DNS leak testing, browser fingerprinting, and identifying mismatches in time zones [27]. To circumvent these detection methods, VPN providers offer features aimed at further obfuscating VPN use, and they are also known to change IP addresses when streaming services like Netflix add them to blocklists [28]. Furthermore, some VPN providers allow individual users to create unique and dedicated IP addresses, making it less likely that these IP addresses will be flagged as being associated with a VPN [29]. Taken together, while VPN detection services are a potentially valuable tool for researchers, more skilled and motivated users can still evade detection.

### Anonymity: Strategies Applied in Future Survey Attempts

To mitigate concerns about anonymity and limitations involving the related strategies, several protocol changes were implemented in our future survey attempts. Each participant’s name, date of birth, and residential mailing address were collected and cross-referenced with corresponding data on LexisNexis’ personal record verification platform. After taking into account the limitations of LexisNexis’ platform, 3 additional protocol decisions were made. First, PO box addresses were not permitted because LexisNexis did not track this information, and someone may own multiple PO boxes. Next, to delay gratification, the first incentive was mailed to the residential address provided rather than sending it electronically. This procedure also ensured individuals who used another person’s identifiable information without their knowledge did not receive the incentive. Third, when self-reported identifiable information did not match the personal record verification platform’s dataset, a picture of a utility bill or driver’s license, or similar identification, was requested, depending on the information that was unable to be verified. This procedure provided participants with a pathway to enroll in the study if they would have been otherwise excluded due to LexisNexis’ imperfect dataset or an honest mistake. IP address–related strategies, including VPN detection services, were not deemed essential given the aforementioned protocol changes. Nevertheless, IP address matching was used to identify multiple survey submissions as a first pass because it was more efficient than conducting a personal record search. Last, participants’ cell phone numbers were requested for authentication purposes and to encourage participants to take the survey from their phones, which provided a more streamlined experience for uploading authentic photos of their devices and liquids. Email addresses were requested as well but not for authentication purposes. Rather, email addresses were collected as a means of contacting participants beyond their cell phone numbers and disseminating gift codes to those who had completed 2 or more surveys.

### Survey Bots

Bots are computer programs that operate on the internet and perform repetitive tasks [30], and survey bots are a type of bot that can submit multiple web-based surveys [31]. Limited information was available on how developers of survey bots design the bots to accomplish their goals; however, a tutorial on YouTube suggested that survey bots are bespoke and adjustable programming scripts [32]. The number of surveys a survey bot submits and the amount of time it takes to complete each survey can be predetermined by the programming script [32]. While the developers can complete surveys quickly and may do so to maximize the number of incentives received, this quick completion rate is not a rule. If the survey bot developer is motivated by the incentive and believes a short survey completion time will result in the survey bot’s detection, they can slow the pace of the survey bot [32]. Furthermore, survey bots can answer different question types, including multiple-choice and open-ended response questionnaire items [32]. For the latter, survey bots can access another service that generates random sentences that are used as a bank of answers for open-ended questionnaire items [32]; however, developers could elect to provide the survey bot with words and sentences that are better aligned with the survey topic to evade detection. Survey bots appear to be an increasingly relevant threat [33]; however, there is a lack of clarity on whether the increasing threat is related to improved reporting, increased prevalence of the issue, or both. Surveys that offer financial incentives are more likely to be targeted by survey bots, although surveys without incentives can also be targeted for practice [33].

### Survey Bots: Mitigation Strategies and Their Benefits and Limitations

CAPTCHA is a web-based tool meant to prevent bots from accessing web pages, web services, and more [34-36]. This tool acts as a gatekeeper by requiring the completion of a task that is easy for humans but difficult for bots [34-36]. The earliest versions involved identifying distorted alphanumeric characters and submitting them in a text field [35,36]. By 2014, machine learning (advanced mathematical) algorithms had become advanced enough that they could solve alphanumeric CAPTCHAs better than humans, which led to image-based CAPTCHAs (eg, selecting all the pictures that contain traffic lights) [35]. However, bots have begun to solve image-based CAPTCHAs at a high rate as well. Sivakorn et al [37] conducted an experiment and found an approach that successfully solved image-based CAPTCHAs more than 70.8% of the time. Furthermore, marketing for CAPTCHA solving services in 2023 suggests such services can use a mix of automated processes (ie, optical character recognition) and humans to solve any CAPTCHA at a 90% to 100% success rate for about US $0.50-US $5.00 per 1000 CAPTCHAs [38-40]. CAPTCHA-solving services also offer application programming interfaces that can be integrated into a survey bot’s script, which enables communications between the survey bot and the CAPTCHA-solving service [38-41]. These findings and available services suggest CAPTCHA is a solvable hurdle for survey bot developers and is not sufficient to prevent survey bots from completing multiple survey submissions, yet CAPTCHA plausibly can deter some developers of survey bots due to the added planning and programming time and modest costs required to circumvent it.

### Survey Bots: Anonymity Strategies Applied in Future Survey Attempts

Despite CAPTCHA’s limitations and the previously discussed strategies for reducing anonymity, we used REDCap’s CAPTCHA task in conjunction with additional checks, such as manually reviewing open-ended responses for repetitious words and phrases and requiring the submission of authentic photos of participants’ most used e-cigarette device. Additional discussion about these strategic choices can be found below in the “Staying Vigilant” section.

### Other Vulnerabilities and Challenges

Other vulnerabilities include lengthy data collection periods that may allow participants more time to devise a strategy for submitting fraudulent surveys, the ability to learn the screening questions and questionnaire easily, nonrandom survey URL text generation, and payment of survey incentives without adequate review of survey submission data quality. Although not directly a vulnerability, one additional challenge is institutional review board (IRB) application language that requires amendments and reports each time a new threat or vulnerability is uncovered.

### Other Strategies Applied in Future Survey Attempts

To mitigate concerns about the duration of the data collection period, we shortened the data collection period as much as possible by increasing the pace of recruitment. Specifically, the catchment area expanded from 3 cities to as many as 404 areas across the United States using Craigslist “jobs” and “gigs” boards. However, a by-product of this decision meant that the puff topography component of the study became a separate and more limited study. To increase the difficulty of learning the screening questions and questionnaire, the “back button” was disabled. Anyone wishing to communicate honest mistakes could notify the research team at the end of the survey in a comment box. Additional mitigation strategies included the randomization of survey URL text, flexible IRB application language that enabled the removal of any participant suspected of misusing the survey, written warnings to participants against survey misuse in the consent form and reminders before the screening questions each wave, and an initial data quality review before incentives were sent to participants. The latter review included monitoring for the use of non-Latin alphabet characters; invalid mailing addresses; multiple submissions from the same individual or household from current or previous waves of data collection; inconsistent responses (eg, age provided in the survey differs from the age provided during registration); minimal number of questionnaire items answered, which was highly unlikely given the skip logic; short completion time; missing attention-checking questions; unusable photos submitted; and remarkably similar open-ended responses across multiple submissions.

## Staying Vigilant

Beyond learning a lesson in the importance of planning mitigation strategies for building a web-based original sample, we also learned the value of staying vigilant as a new threat and vulnerability emerged after commencing with wave 1 data collection (ie, the first wave of data collection after the initial survey attempt that failed).

At the start of wave 1, participants still had the optional ability to submit photos of their most used e-cigarette device to allow for the accurate collection of technical details, which can be difficult to self-report. They were intended to be authentic photos of their device rather than images downloaded from the internet. Initially, this worked as expected, with only a handful of survey submissions including images downloaded from the internet, which we attributed to participants not having their devices readily available. However, approximately 2 months into the relaunch of wave 1, there was a sudden increase in device images that appeared to be downloaded from the internet (34 images over 3 days, of which many were identical images). Out of an abundance of caution, we temporarily suspended the delivery of gift codes to investigate the cluster of survey submissions more thoroughly.

Upon closer examination, other issues with these survey submissions were discovered. Notably, open-response answers were remarkably similar, with all the suspicious survey submissions providing nearly identical responses to the inquiry for feedback to improve the survey. These included responses such as “good,” “GOOD,” “very good,” “VERY GOOD,” “well,” and “very well.” In addition, self-report device brand and model were limited to only a few responses (eg, “JUUL,” “Vaporesso,” “Luxe,” “Voopoo,” and “Drag”), which aligned with the examples provided in the questionnaire item. Of further concern, these submissions passed data quality and personal record checks and there were no obvious connections between the individuals who appeared to submit the suspicious surveys (ie, there was no geographic pattern observed from the addresses provided, and reported ages also widely varied).

Discerning precisely how these suspicious survey submissions were submitted and evaded detection is difficult. Plausibly, someone developed a survey bot capable of circumventing the CAPTCHA and submitted multiple surveys, although, equally plausibly, a single individual or a group of individuals who are somehow connected could have repeatedly attempted the survey without the aid of a survey bot [10]. Presuming that the culprits were not a group of individuals operating from a written script, the idea that a survey bot developer or other individuals involved passed the personal record check is perplexing. One possibility is that they read the consent form, recognized we were authenticating participant identities, and used a database with authentic identifying information. While unlikely, we also cannot dismiss the possibility that these data and circumstances were a coincidence.

The motivating factors for the individuals responsible for the suspicious survey submissions remain unclear as well, although the motivation could have been to create chaos or, more likely, financial gain [5,8-10]. Assuming a database with authentic identifying information was used, the individuals responsible would not have received the initial gift code for the baseline survey, as the first gift code was delivered by mail to the address provided. This detail may have been overlooked by the individuals; however, they may have recognized from the consent form that future gift codes would be delivered electronically and could potentially profit from these efforts in subsequent waves. A similarly patient approach to survey fraud has been taught by at least 1 website dedicated to training individuals to fraudulently submit a large volume of surveys on their own: “...just create enough accounts and wait for the right opportunity. You can in just 1 day, make enough money to last you for several months” [10].

Ultimately, we decided that the cluster of submissions was suspicious enough to warrant being dropped from the dataset, and 3 actions were taken to mitigate the chances of recurrence. First, we required photo submissions of each participant’s most used device to ensure that all were in possession of an e-cigarette. Second, all photo submissions were reviewed as part of the initial data quality review to ensure they were not professional, downloaded, or screenshot images; duplicates; remarkably similar to photos already submitted (eg, distinctive marks, lighting, and background objects); an object other than an e-cigarette; taken from a store; or inconsistent with the self-reported brand (for brands that were listed as examples in the corresponding questionnaire item only). Finally, all open-ended responses were reviewed once a week as part of the initial data quality review to ensure that repetitive open-ended answers were not submitted. These strategic choices further raised the bar required to deceive our team. For example, developers of survey bots or others attempting to deceive our team in the future would be required to purchase or access e-cigarettes, take authentic photos of the e-cigarettes in different lighting environments, and submit nonrepetitive and logical open-ended responses.

## Reflections 4 Years Later

Four years after our unsuccessful attempt to recruit participants for a web-based survey, we successfully created a cohort and completed 5 survey waves of data collection between May 2020 and April 2023 (wave 1: n=1179, wave 2: n=1187, wave 3: n=1219, wave 4: n=1224, and wave 5: n=1290). After implementing additional mitigation strategies in response to the suspicious survey submissions in wave 1, no new threats or vulnerabilities were uncovered (Textbox 2). In addition, although we cannot dismiss the possibility that the final dataset contains a few fraudulent survey submissions, there is little to no evidence that the dataset’s integrity has been compromised to a degree that it would bias the sample and analyses. For example, the sample of those who frequently use e-cigarettes primarily consists of people who self-reported daily use (wave 1: 1081/1179, 91.7%; wave 2: 1104/1187, 93.0%; wave 3: 1154/1219, 94.7%; wave 4: 1170/1224, 95.6%; and wave 5: 1234/1290, 95.7%), which is demographically similar to respondents using e-cigarettes daily from the 2019 Tobacco Use Supplement to the Current Population Survey [42], with the exceptions that participants in the VAPER Study sample were relatively younger and had lower incomes. Moreover, often the VAPER Study findings have been consistent with the broader literature and policy context. For example, participants’ most used flavor for their most used device included a wide range of flavors for all device types except disposable pod devices (eg, JUUL). Specifically, 93.4% (156/167) of those mostly using disposable pod devices during wave 5 indicated that their most used flavor was tobacco or menthol, which largely is consistent with expectations given a US Food and Drug Administration regulation that only allows the sale of tobacco- and menthol-flavored pods [43,44].

**Risk mitigation strategies used in the VAPER (Vaping and Patterns of E-cigarette Use Research) Study, waves 1-5.**

**Presurvey strategies:**

- Requested flexible institutional review board protocols for easy removal of participants suspected of misuse

**In-survey strategies:**

- Authenticated cell phone numbers
- Collected IP addresses
- Used CAPTCHA (Completely Automated Public Turing test to tell Computers and Humans Apart)
- Shortened the data collection period
- Generated random URLs for accessing the survey
- No back button
- Required photo submissions of their most used e-cigarette
- Used written warnings to warn against misuse in the consent and before the screening questions

**Postsurvey strategies:**

- Authenticated names, dates of birth, and residential mailing addresses
- Requested identification cards and utility bills, as needed
- Mailed incentives to physical addresses
- Completed an initial data quality review prior to sending incentives

The study protocols, inclusive of the mitigation strategies, were adequately scalable and cost-effective given the project resources available [14]. One of the more significant expenses unique to studies with an original sample was the Craigslist postings to advertise the survey. These costs peaked at US $53 per valid participant in wave 2 (covering 150 Craigslist areas over 4 months to replace those lost to follow-up). Costs decreased to US $6-US $10 per valid participant in waves 3-5 (covering 404 Craigslist areas over 2-2.5 months to replace those lost to follow-up). These costs decreased in response to the number of Craigslist areas increasing (individual Craigslist areas can become more depleted over a period of a few months), a larger proportion of the posts being on the “gigs” boards (these posts are cheaper than those on the “jobs” boards), and improved reposting schedules (more populated areas require more frequent reposting). In terms of mitigation strategies, several were implemented at minimal or no cost, as follows: (1) authenticating phone numbers, (2) using CAPTCHA, (3) generating random URLs for survey access, (4) prohibiting the use of the back button, (5) requesting flexible IRB protocols to easily remove participants suspected of misuse, (6) including written warnings against misuse in the consent and before the screening questions, and (7) requiring photo submissions of participants’ most used e-cigarettes. Other mitigation strategies entailed significantly higher costs in terms of subscription fees. For example, an annual subscription to LexisNexis’ personal record verification platform costs US $4876 for access by 3 users, as of March 2020 (prices may vary and could be outdated). The most substantial and difficult-to-quantify cost was the staff effort required to (1) post and repost Craigslist postings, (2) use the LexisNexis’ personal record verification platform, (3) follow up with participants who could not be authenticated using LexisNexis’ platform, (4) conduct an initial data quality review prior to sending incentives, (5) mail incentives to physical addresses, and (6) conduct data cleaning and analysis. Moreover, staff effort was essential in planning mitigation strategies ahead of data collection and swiftly implementing postsubmission mitigation strategies to ensure a positive participant experience. For example, after our investigation into the initial survey attempt that failed in 2019, preparation and ethics approval for the relaunched wave 1 took approximately 9 months, and starting with wave 1, all incentives were sent within 2 weeks of survey submission to participants who had their identities authenticated and passed the initial data quality checks.

As previously highlighted by our experiences and the learnings from the literature, the specific tactics used by individuals attempting to submit fraudulent surveys can be dependent upon mitigation strategies in place, but, likely, study design and the intended population (eg, lower prevalence vs higher prevalence) play important roles as well. The methods and array of related strategies highlighted in this paper were successful for the VAPER Study’s study design and population, but a similar approach may be insufficient for other study designs and populations and may be excessive for others. For instance, we initially paid participants US $10 to complete a 20-minute survey and included screening questions intended to only include a lower prevalence population. For the financially motivated, this payment schedule may have provided a financial incentive to learn the screening questions through multiple attempts in order to complete one or multiple fraudulent survey submissions. This possibility is consistent with the work of Bowen et al [8], who found that participants eligible for an incentive were 6 times more likely to submit more than one survey. Furthermore, general population studies likely will recruit participants more quickly than studies recruiting a lower prevalence population, resulting in less time to develop bespoke survey bots or to implement other strategies. Thus, general population studies may encounter fewer threats, although this is an empirical question that awaits further investigation.

Nearly all the mitigation strategies implemented in the VAPER Study can be used by researchers in other medical and public health fields seeking to strengthen their web-based original samples, resources permitting. However, requiring photo submissions is not an appropriate strategy for all studies. For the VAPER Study, the required submission of device photos provided us with added confidence that the survey submissions came from the target population. A similar strategy can be used for other web-based surveys that recruit populations likely in possession of an item, such as people who frequently use combustible tobacco products or alcohol (eg, photos of product packaging), who take a specific medication (eg, photos of the bottle containing the medication), or who work in a specific occupation (eg, photos of an identification badge from their employer). The strategy is less appropriate in instances where the target population is less likely to be able to submit photos of the desired item. For example, people who infrequently use combustible tobacco products or alcohol may not be in possession of a product package at the time of the survey. Additionally, this strategy is not applicable when there is no physical item uniquely associated with all individuals in the target population (eg, members of a religious group). In such cases, open-ended questions that solicit information likely to be commonly known by the target population—but not by others—such as colloquial neighborhood names in city-specific studies, may be a useful alternative [45]. However, the efficacy of this approach remains uncertain, as specific information commonly known to a target population can still be searched for by those attempting to submit fraudulent surveys.

While we are satisfied with the integrity of the VAPER Study dataset, we recognize that the recruitment and mitigation strategies used likely introduced at least some sampling bias. Regarding recruitment strategies, the sample was drawn from the internet, where use is lower among individuals aged 65 years and older (88%) compared with younger age groups (96%-98%) [46]. Additionally, our web-based recruitment predominantly targeted the “jobs” and “gigs” boards on Craigslist, where individuals are often seeking income-generating opportunities. We suspect that these factors likely contributed to a younger, lower-income sample compared with the Tobacco Use Supplement to the Current Population Survey. Recruitment from other websites or social media platforms may have yielded a sample with a different level of representativeness. Address-based sampling using mailed survey invitations with an access code may have resulted in a more representative sample and should be considered for future studies [47]. In terms of mitigation strategies, privacy concerns may have deterred some individuals from participating due to potential data security risks, which are inherent to both in-person and web-based studies that require identifiable information for enrollment. Furthermore, American adults who could not write in languages using the Latin alphabet were excluded given our experience with the failed survey attempt in 2019.

Overall, web-based recruitment and web-based survey data collection proved to be more challenging than we anticipated, but much has been learned from our initial survey attempt that failed and subsequent successes. As a result, we have reasonable confidence in the integrity of the data and a stronger understanding of their strengths and limitations. We encourage other researchers conducting web-based surveys to consider the threats and vulnerabilities that could impact their data integrity, to incorporate appropriate mitigation strategies, and to develop effective monitoring procedures. In addition, we encourage more researchers to report their challenges and successes and to report additional details about their web-based survey methodologies for mitigating the submission of fraudulent surveys and preserving data integrity in their manuscripts.

## Abbreviations

CAPTCHA: Completely Automated Public Turing test to tell Computers and Humans Apart
IRB: institutional review board
LGBTQ+: lesbian, gay, bisexual, transgender, queer
PO: post office
REDCap: Research Electronic Data Capture
VAPER: Vaping and Patterns of E-cigarette Use Research
VPN: virtual private network

## Data Availability

The datasets for waves 1-5 generated during this study are available from the corresponding author on reasonable request and after a review of the proposed research and qualifications of the applicant.
